# Subjective sleep quality in association with psychosocial resources and stressors in Germany – an exploratory data analysis of the NRW Health Survey 2024

**DOI:** 10.25646/14373

**Published:** 2026-09-09

**Authors:** Daria Kozica*, Anja Viehmann*, Jonas Weidtmann, Brigitte Borrmann

**Affiliations:** State Office for Health and Occupational Safety North Rhine-Westphalia, Health Reporting Unit, Bochum

**Keywords:** Sleep quality, Adults, Germany, Depression, Loneliness, Mental health, Protective factors

## Abstract

**Background:**

To date, numerous factors that affect sleep quality and duration have been identified, including physical and mental health conditions. However, there is an urgent need for effective approaches to counteract the rising prevalence of poor sleep quality.

**Methods:**

As part of a representative population survey (NRW Health Survey 2024), data were collected from 2,002 adults in North Rhine-Westphalia (NRW), Germany, on sleep quality, health status, substance use, and individual resources and stressors.

**Results:**

Poor sleep quality affected 36.9 % of the participants. In the logistic model, positive associations were observed between subjectively poor sleep quality and, among other factors, being female, depression/depressive moods, and increased levels of loneliness. A sense of autonomy and competence appear to be protective factors.

**Conclusion:**

Associations between poor sleep quality and depression, and between psychosocial resources and stressors, suggest that mental health is a key factor in determining the underlying causes of poor sleep quality. Possible interactions should also be taken into account. In particular, strengthening psychosocial resources should be given greater attention at all levels of prevention.

**Key messages**

► In North Rhine-Westphalia, 36.9 % of adults (43.9 % of women and 31.4 % of men) report poor sleep quality.
► Among those with a low socio-economic status, 47.5 % are affected by poor sleep quality.
► The highest prevalence is observed among men who have experienced depression or depressive moods in the last 12 months (74.6 %) and women who experience high levels of loneliness (71.4 %).
► Among adults with depression or depressive moods, poor sleep quality was 2.4 times more common than among those without depression or depressive moods.
► The fulfilment of basic psychological needs is associated with better subjective sleep quality.

## 1. Introduction

Adequate sleep duration and good sleep quality have a positive effect on physical and mental health as well as quality of life [1, 2] . A meta-analysis incorporating 51 studies identified associations between sleep disorders and type 2 diabetes, cardiovascular diseases, Alzheimer’s disease, Parkinson’s disease and various mental health conditions. Possible pathomechanisms include, for example, increased inflammatory processes and oxidative stress caused by sleep deprivation [3]. Good sleep is therefore a significant health resource, but appears to be increasingly difficult for more and more people to achieve. This is evident from the increased prevalence of various sleep disorders and the common use of sleeping pills [4–6]. The public health relevance of this issue arises from the widespread prevalence of sleep problems in the population (see Discussion) and the high direct and indirect healthcare costs associated with sleep disorders [7].

Numerous factors can affect night-time sleep. In addition to environmental factors such as noise, temperature and light exposure, individual resources and stressors (e.g. shift work, everyday stress and long-term worries) as well as lifestyle (media consumption, diet, exercise, alcohol and tobacco use, sleep hygiene, etc.) have an impact on sleep duration and sleep quality [8]. Physical and mental health conditions can also impair sleep, leading to a process reinforcing itself. For example, research has shown that depression can increase the likelihood of experiencing daytime sleepiness and sleep disorders [9–12]. Conversely, numerous cohort studies demonstrate that sleep disorders can contribute to depression [13] while intervention studies suggest that treating sleep disorders can alleviate depressive symptoms [14].

To date, little attention has been given to the examination of psychosocial resources in relation to sleep quality. However, focusing on protective factors in this regard can highlight aspects that go beyond mitigating known risk factors, thereby broadening the spectrum of preventive measures and therapeutic approaches. 

A significant health-related resource lies in the fulfilment of fundamental psychosocial needs for autonomy, competence and relatedness. These three components form the core of the Self-Determination Theory (SDT) developed by Ryan and Deci [15]. This motivational psychological approach posits that fulfilling universal basic needs is crucial for intrinsic motivation and well-being. Various studies have shown that perceived fulfilment of these needs predicts general well-being similarly across cultures [16–18] relatedness, autonomy, and competence. SDT shares conceptual links with concepts from developmental and health psychology, such as self-efficacy, locus of control, salutogenesis (sense of coherence) and resilience, as well as with positive psychology. One common feature is the resource-oriented focus of these concepts. 

Under favourable social conditions, the fulfilment of the basic needs for autonomy, competence and relatedness is more likely. By contrast, living conditions characterised by social disadvantage are more frequently associated with experiences of being controlled by others, helplessness and social exclusion, and thus with stress [19, 20]. The application of SDT and similar concepts can therefore help to shed light on mechanisms behind differences in health outcomes between various population groups and trends over time in the context of societal changes, and to develop effective prevention strategies.

This paper reports on the prevalence of perceived sleep quality in the population of North Rhine-Westphalia (NRW), based on the NRW Health Survey 2024. Furthermore, it explores various stressors and resources in order to identify specific prevention needs. 

## 2. Methods

### 2.1 Study design and sample

The standard questions in the regular, representative NRW Health Surveys include, among others, questions on health behaviour and subjective health status. In addition, question modules on varying focal topics are used. The focal topic of the NRW Health Survey 2024 was mental health.

The population for the survey comprises German-speaking adults living in private households in North Rhine-Westphalia. From this population, a total of 2,002 people were surveyed for the NRW Health Survey 2024. The survey was conducted by a polling organisation using a standardised questionnaire in the form of computer-assisted telephone interviews (CATI) between 30th August and 7th October 2024. 

The selection of survey participants was based on a combined landline and mobile phone sample, with mobile numbers accounting for 40 % of the sample. In the landline sample, selection was carried out using a stratified, multi-stage random sample based on the ADM telephone sampling system. Where there were several people aged 18 years or older in a household, the person to be interviewed was selected using the ‘last birthday’ method. No interviews were conducted with substitute respondents from the selected households. The selection of respondents in the mobile phone sample was also carried out using a multi-stage random sampling method based on the mobile phone selection frame established by the ADM’s Sampling Working Group which is updated annually. The selection universe comprises all individuals in the population who can be reached via a mobile phone. As regional filtering involves a significant expenditure of resources, a targeted mobile phone sample was formed in North Rhine-Westphalia from participants in daily, representative surveys who were willing to be re-interviewed. With a net sample of 7,246 individuals, a response rate of 27.6 % was achieved. In 47 % of cases, the target individual could not be reached; 24 % of those contacted refused to take part; and 2 % discontinued the interview. 

Resulting from the study design described above, people living in care homes and those without sufficient knowledge of German could not be included in this cross-sectional survey. Furthermore, willingness to participate varies across different population groups. In particular, people with a low level of education are less likely to be willing to take part in telephone surveys.

### 2.2 Statistical methods

The participants’ responses were analysed descriptively, and group differences were examined using the chi-squared test, t-test and Mann-Whitney U test, respectively. Stratification was carried out dichotomously according to reported sleep quality (poor vs. moderate to good) and gender. A p-value of < 0.05 was considered statistically significant. 

Multiple logistic regression was used to examine psychosocial resources and stressors as potential predictors on sleep quality in order to answer the research questions. The analyses were exploratory. To derive the final model, (1) an association diagram was created to illustrate the relationships between the variables of interest, based on the current literature. In addition, (2) a correlation matrix was calculated. Using the information from these two steps, the variables for model building were determined. These included general health status, chronic diseases, psychological stress, satisfaction with paid and unpaid work, stress arising from responsibility for others (e.g. children or care recipients), perceived social support, loneliness, interpersonal trust, basic psychological needs, satisfaction with the residential environment, perceived safety in the residential environment in the dark, smoking status and alcohol consumption, as well as socio-demographic variables (gender, age, migration status, level of formal education, income and derived socio-economic status). 

Following the calculation of univariate logistic models (3) and analyses using a full model (4), model fit criteria were applied in the final step (5) to determine the final analytical model. Odds ratios (OR) for ‘subjectively poor sleep quality’ were calculated, along with 95 % confidence intervals (CI), for all participants with valid values for the variables included in the final model. Calculations were carried out for the overall population, as well as stratified analyses by gender (female/male). All analyses were carried out using SPSS version 27.0.1.0.

### 2.3 Instruments

#### Sleep quality

The outcome – subjective sleep quality – was assessed using the German version of the Athens Insomnia Scale for Non-Clinical Application (AIS-NCA) [21, 22]. This scale consists of seven items and covers the areas of falling asleep, staying asleep and daytime sleepiness over the past four weeks. The response options are rated on a Likert scale from 0 to 4 and added together to give a total score (range 0 to24 points). The total score was dichotomised in the same way as for the Athens Insomnia Scale [23] and adapted for the German scale. A total score of ≥ 13 indicates that there has been an impairment in daytime functioning and sleep problems, and thus poor sleep quality, over the past four weeks.

#### Psychosocial resources and stressors

##### Health status and chronic diseases

Through a self-assessment, participants were asked to report their general state of health, with the response options ‘very good’, ‘good’, ‘fair’, ‘poor’ and ‘very poor’. The question regarding the presence of chronic diseases was dichotomous (yes/no).

##### Psychological stress and depression/depressive mood

Perceived stress levels were measured using a single item (‘To what extent do you feel stressed by everyday life?’) on a five-point scale (‘very much’ to ‘not at all’). In addition, responsibility for the care of minor children living in the household and the provision of informal care at home were assessed. Further sources of stress were assessed through questions on satisfaction with unpaid work and personal and/or occupational stress arising from responsibility for other people over the past four weeks. 

Participants who reported that they had ever been diagnosed with depression or depressive moods by a doctor or psychotherapist were asked in more detail whether this had also been the case in the last 12 months. This variable was included in the analyses as a dichotomous variable (yes/no).

##### Loneliness

To measure the level of loneliness, the University of California Los Angeles Loneliness Scale (UCLA-LS) was used, comprising three items, each rated on a five-point scale from 1 (never) to 5 (often). The total score is aggregated and averaged. A score > 3 is classified as ‘increased loneliness’, whilst ≤ 3 indicates ‘no increased loneliness’.

##### Interpersonal trust

Interpersonal trust refers to ‘the expectation of an individual or a group that they can rely on the words and promises, or verbal or written statements, of others or a group’ [24]. Interpersonal trust was measured using the five-point KUSIV-3 scale, aggregated into a total score and averaged. A high score indicates high interpersonal trust [25].

##### Social support

The Oslo-3-Item Social Support Scale (Oslo-3 scale) was used to measure social support. The results were aggregated into a total score for the analyses (range 3 to 14) [26, 27]. Poor support is assumed for a score of 3 to 8 points, moderate support for a score of 9 to 11 points, and high support for a score of 12 to 14 points.

##### Basic psychological needs

The fulfilment of basic psychological needs was assessed using the German 12-item version of the Basic Psychological Need Satisfaction and Frustration Scale (BPNSFS). The questionnaire is based on the Self-Determination Theory developed by Ryan and Deci [15]. According to this approach, autonomy (the feeling of being the agent of one’s own actions), competence (the feeling of being effective) and relatedness (the feeling of being understood and cared for by others) are among the central, cross-cultural basic psychological needs. The short scale consists of four questions for each of the three basic needs mentioned. The questions can be answered on a five-point scale (‘strongly agree’ to ‘strongly disagree’). The scores for each subscale were summed and averaged. A high score reflects a high degree of fulfilment of the respective basic need. These variables were included as continuous variables. 

##### Housing and the local environment

As part of the NRW Health Survey 2024, items relating to satisfaction with one’s flat or house, place of residence (town or municipality) and the immediate living environment were collected and could therefore also be included in the context of the present research question. As the main objective of the survey was not to investigate the relationships between sleep quality and potential contributing factors, questions regarding exposure to noise and light were not included. 

Participants’ responses were recorded on a scale from 1 (very satisfied) to 5 (very dissatisfied). The sense of safety in one’s own neighbourhood in the dark was assessed using a single item on a four-point scale ranging from 1 (safe) to 4 (unsafe). In addition, the frequency of threats or attacks (affecting oneself or third parties) in the neighbourhood over the past 12 months was recorded.

#### Health behaviours 

##### Smoking

The participants’ smoking habits were assessed using the following question: ‘Do you currently smoke every day, do you smoke occasionally, have you given up smoking, or have you never smoked, even occasionally? E-cigarettes are not included in this.’. Two groups were compared for the analysis: (1) regular (daily) smokers and (0) irregular (non-daily) smokers, non-smokers or former smokers.

##### Alcohol consumption

To assess alcohol consumption, the Alcohol Use Disorder Identification Test-Consumption (AUDIT-C) [28], consisting of three questions, was used: the minimum AUDIT-C total score is 0 and the maximum score is 12. According to the AUDIT-C, once the total score had been calculated, scores of ≥ 4 for women and ≥ 5 for men were classified as risky consumption (1). These were dichotomised in comparison with individuals who drink moderately or never (0). 

#### Sociodemographics

##### Gender and age

Gender was assessed using the item ‘What is your gender?’. Possible responses were female, male and non-binary. In the analyses stratified by gender and in the logistic model for the overall group, cases categorised as ‘non-binary’ were excluded due to the small number (n = 5). Participants were asked for their year of birth. Their age at the time of the survey was calculated from this, and the following age groups were formed: 18 – 34 years, 35 – 49 years, 50 – 64 years and 65 years and over.

##### Socio-economic status (SES) and education

Data on attainment in formal education, vocational training, occupational status and net household income were collected using standardised instruments. An index was constructed in line with the approach used in the GEDA study: when building the model, socio-economic status was included as an index, whilst only educational and occupational qualifications were included according to the CASMIN classification (Comparative Analyses of Social Mobility in Industrial Nations) [29].

##### Migration history

Migration history was defined in accordance with the definition set out in the Act on the Promotion of Social Participation and Integration in North Rhine-Westphalia (TIntG NRW) [30]. This includes persons without German citizenship as well as Germans who immigrated to the present-day territory of the Federal Republic of Germany after 1955, or at least one of whose parents immigrated after 1955.

## 3. Results 

The net sample comprised 7,246 individuals (100 %). Of these, 1,722 people (23.8 %) refused to take part, 144 people (2.0 %) abandoned the interview, and 3,378 people (46.6 %) could either not be reached or were only reached via an answering machine, or an interview was not possible during the fieldwork period. 2,002 participants (27.6 %) successfully completed the interview, including 1,130 men (56.4 %), 867 women (43.3 %) and five people who identified as ‘non-binary’ (0.3 %). 203 participants were excluded from the present analyses due to missing or incomplete data. Table 1 shows the characteristics of the study population for which the final model could be calculated.

The prevalence of subjectively poor sleep quality according to socio-demographic and health variables is shown in Table 2. Overall, 36.9 % reported subjectively poor sleep quality, with the proportion being higher among women (43.9 %) than among men (31.4 %). 

In the general population, the experience of subjectively poor sleep quality varies according to age, socio-economic status, satisfaction with unpaid work, the presence of depression or depressive moods, and levels of loneliness. Among those who had experienced depression or depressive moods in the past 12 months, 70.2 % reported poor sleep quality, compared with 33.1 % of those who had not experienced depression or depressive moods in the past 12 months. Among women, differences were observed with regard to age, socio-economic status, depression or depressive mood in the past 12 months, and levels of loneliness. Among men, significant differences were observed with regard to socio-economic status, satisfaction with unpaid work, depression or depressive mood in the past 12 months, and levels of loneliness.

A comparison of the overall mean scores (Table 3) for the groups with poor sleep quality and moderate to good sleep quality, with regard to the components of basic psychological needs and interpersonal trust, shows that the groups differ statistically. 

The results of the univariate models relate to the analysis population in the final model. In the univariate models, (very) high levels of everyday stress, a (very) poor subjective state of health, low levels of social support, increased feelings of loneliness, interpersonal trust, depression or a depressed mood in the past 12 months, a (somewhat) unsafe feeling in the dark within the home environment; female gender; low and medium socio-economic status; dissatisfaction with unpaid work, one’s flat or house, and place of residence; and the three dimensions of basic psychological needs – autonomy, competence and relatedness – were associated with poor sleep quality. The 50 to 64 years age group is the most severely affected by sleep problems compared with the other age groups. All other variables examined showed no association or only very weak associations (Supplementary Material Table 1).

Variables that exhibited multicollinearity were not included in the final model. In such cases, the variable with the higher OR was included in the model. 

The remaining 11 variables (gender, age, socio-economic status, smoking, satisfaction with unpaid work, depression/depressive mood, loneliness, interpersonal trust and the components of basic psychological needs) were included in the multivariate model because a statistical association was evident, they could contribute to explaining the model, or they represented a confounding factor. The three components of basic psychological needs, which were correlated with one another, were all included in the final model, as they represent parts of an overall construct.

The overall model reveals associations between female gender, depression/depressive moods, unmet basic psychological needs for autonomy and competence, and poor sleep quality (Supplementary Material Table 2). Low socioeconomic status, being aged between 50 and 64, and increased levels of loneliness are only marginally significantly associated with poor sleep quality.

**Figure 1:**
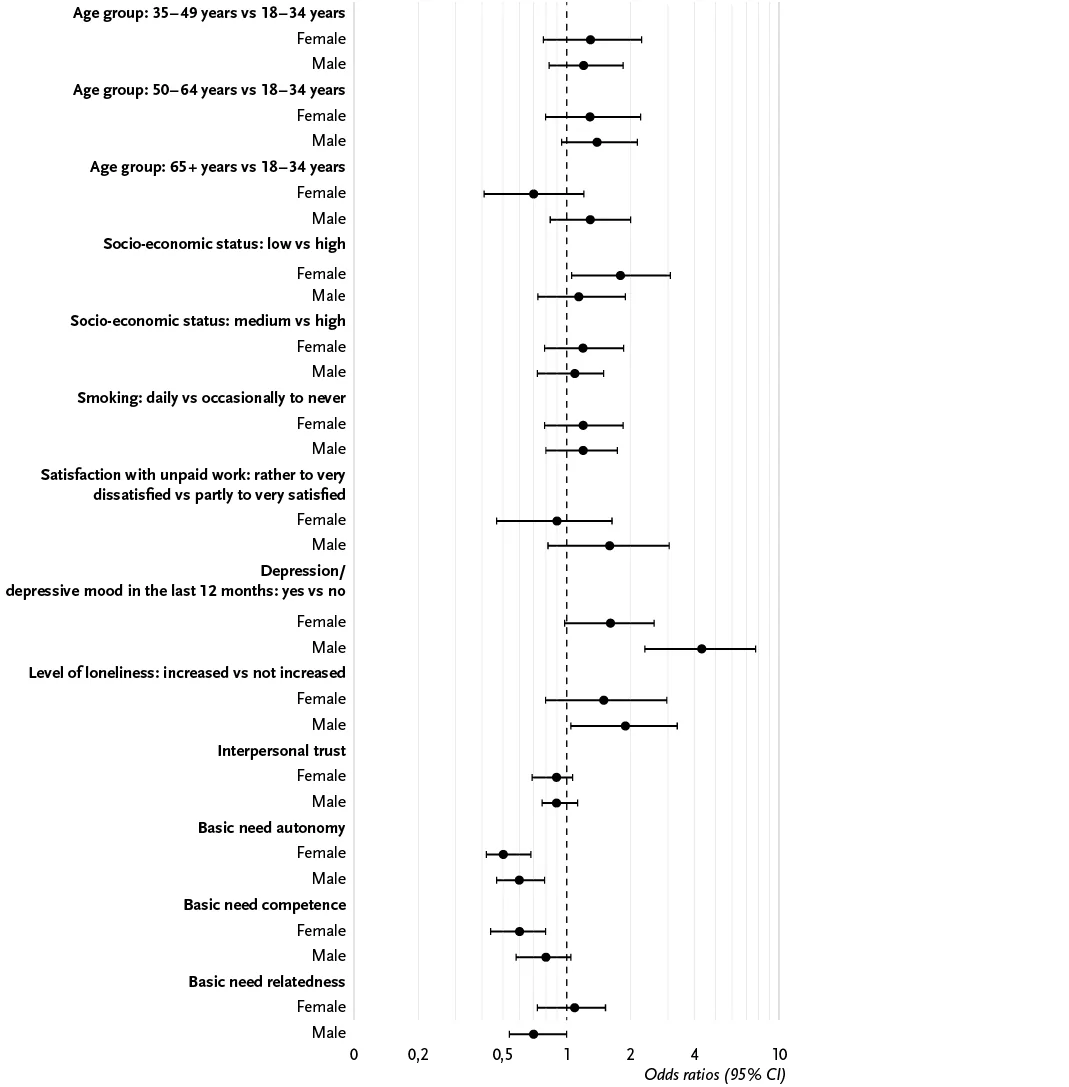
Odds ratios and 95 % confidence intervals for the multivariate binary logistic regression analyses of subjectively poor sleep quality (n = 781 women, n = 1,018 men), stratified by gender. Source: NRW Health Survey 2024

The stratified analysis reveals a strong association between low levels of autonomy and competence and poor sleep quality among women (Figure 1, Supplementary Material Table 2). Low socio-economic status is also marginally associated with sleep quality in women. In the stratified analysis, a link between depression/depressive mood, loneliness and poor sleep quality is found only in men. Unlike among female participants, autonomy is the only basic psychological need significantly associated with sleep quality among male participants. However, a trend can be observed regarding the basic need for relatedness: men whose need for relatedness are not met are more likely to experience poor sleep quality. This trend is not observed among women. 

## 4. Discussion

The presented results from the exploratory analyses using data from the NRW Health Survey 2024 show that a total of 36.9 % of participants reported poor subjective sleep quality over the past four weeks. The prevalence is higher among women than among men. 

In the overall model, 11 of the variables (n = 26) considered on the basis of the literature review were included in the main model. Among the potential predictors, depression/depressive mood, loneliness, the unfulfillment of basic psychological needs and low socio-economic status were found to be associated with poor sleep quality in the overall population. When stratified by gender, the following differences emerge: for men, the association with the variables depression/depressive moods and loneliness persists. For women, an association with low socio-economic status is also evident in the overall model. 

In contrast to female participants, poor sleep quality in male participants is only associated with an unmet basic need for autonomy. However, men with a deficient sense of relatedness are more likely to experience poor sleep quality. This trend is not observed among women. 

The prevalence of poor sleep quality observed in the population of North Rhine-Westphalia is comparable to that found in another German population-based study (n = 9,380) [5], in which a prevalence of 36 % was determined. Kolip et al. report a prevalence of 19.6 % among 18- to 31-year-olds based on data collected between 2014 and 2017 [31]. In the younger population of North Rhine-Westphalia, significantly higher prevalences are observed in the NRW Survey 2024 (34.2 % among 18- to 34-year-olds). According to Beyer et al. (2026), almost one in three Germans is affected by difficulty falling asleep or staying asleep, with difficulty staying asleep (31.7 %) being reported more frequently than difficulty falling asleep (16.3 %) [32]. According to analyses by a large German health insurance (AOK), the prevalence of physically caused sleep disorders tripled between 2004 and 2023. A seven-fold increase has been observed for psychologically induced sleep disorders [33].

Both in the population-based study involving participants aged between 18 and 80 and for the cohort of young adults, differences regarding gender and social determinants (education, employment status and socio-economic status) are reported, as is the case for the population of North Rhine-Westphalia [34].

A study of university students in the USA revealed a link between the fulfilment of basic psychosocial needs and both the weekly sleep duration and sleep quality [35]. A Belgian study involving patients with chronic fatigue also concluded that unmet needs for autonomy, competence and relatedness correlate with stress levels, and lead to increased fatigue and poorer sleep quality [36]. A reciprocal relationship is also conceivable, as sleep problems can impair the fulfilment of basic needs in the long term, as shown by two studies involving adolescents [37]. 

Despite a substantial increase in the number of studies on sleep quality in recent years, there remains a need for systematic research, particularly into the interactions between subjective sleep quality and specific psychosocial factors. Our analyses of stress levels and sleep quality reveal a positive association and follow the same pathway as the components of basic psychological needs. In the field of clinical sleep research into non-organic sleep disorders (insomnia), the concept of hyperarousal is now firmly established [38]. Hyperarousal describes a state that is frequently observed in people with insomnia. At the cognitive level, it manifests as constant rumination and an inability to switch off mentally. Emotionally, it leads to a subjective sense of inner restlessness as well as strong negative feelings towards sleep deprivation. At the autonomic level, hyperarousal manifests through physical stress responses such as palpitations and sweating, whilst at the motor level it is reflected in general physical tension. Exactly how basic needs relate to subjectively perceived sleep quality requires further scientific investigation. However, it seems plausible that the unfulfillment of basic needs such as autonomy (self-determination), a sense of competence and relatedness leads to stress reactions, which in turn could exacerbate persistent overexcitement.

Numerous studies have demonstrated that people affected by depression frequently suffer from sleep disturbances [8–11]. This link appears plausible, because a tendency to ruminate is one of the symptoms of depression and can, at the same time, cause sleep problems [39, 40]. Similarly, there are reciprocal links between loneliness and sleep disturbances [41]. 

A study of data from the UK Biobank showed that, in a clinically diagnosed sample of patients with depression, sleep health variables were associated with the course of depressive moods and loss of interest [42].

The identified links between the failure to fulfil basic needs for autonomy and competence and the presence of poor sleep quality may show that a lack of a sense of autonomy and competence generates or exacerbates stress, for example because those affected tend to feel helpless in the face of everyday stress or in crisis situations. This also can undermine the motivation to adopt healthy sleeping habits. The concept draws attention to environmental factors that contribute to the fulfillment of basic psychosocial needs.

The data used were not collected specifically for this research question, which restricts the scope of the analysis. Other relevant factors, such as the sleeping environment, shift work, environmental noise, and the use of sleeping pills and other medication that may affect sleep, were not recorded. However, as the NRW Health Survey collected a wide range of variables, it was possible to examine variables from different domains. Due to the cross-sectional design, no conclusions regarding causality can be drawn.

A further limitation is that sleep quality was assessed via self-report rather than through sleep laboratory analyses or comparison with medical diagnoses. However, the use of a questionnaire designed for non-clinical settings makes it possible to identify subclinical sleep problems as well. The data collected consists of self-reported information. This may have led to distortions due to social desirability, particularly in relation to questions concerning substance use or mental health conditions and problems.

Certain population groups are not represented, or are under-represented. Both individuals with low and those with high socio-economic status are under-represented in the sample. Individuals without sufficient knowledge of German were unable to take part in the NRW Health Survey. Furthermore, serious health problems may also act as a barrier to participation in telephone interviews.

Although the number of studies on sleep quality has increased significantly in recent years, there is still a demand for systematic research into the relationship between subjective sleep quality and psychosocial factors. In particular, it remains unclear to what extent psychosocial resources can moderate or compensate for the negative effects of stress, taking socio-demographic characteristics into account. Furthermore, it remains unclear which psychosocial factors have a particularly strong impact on sleep quality and how these findings can be utilised for prevention and health promotion. 

However, based on current evidence, the systematic consideration of basic psychosocial needs within the context of designing health-promoting environments – such as nurseries, schools, workplaces and care homes – should help to reduce stress factors and strengthen individual resources. This includes, for example, reliable relationship structures and protection from neglect, violence and stigmatisation (relatedness), opportunities for participation and influence (autonomy), as well as appropriate challenges and a culture of feedback (sense of competence). 

Given the high prevalence of sleep disorders and the associated health risks, this issue is of crucial importance in terms of health policy.

## Figures and Tables

**Table 1:** Description of the study population (n = 1,799). Source: NRW Health Survey 2024

	**n**	**%**
**Gender**		
Female	781	43.4
Male	1,018	56.6
**Age group (in years)**		
18 – 34	371	20.6
35 – 49	494	27.5
50 – 64	520	28.9
65 +	414	23.0
**Socio-economic status**		
Low	320	17.8
Medium	1,106	61.5
High	373	20.7
**Smoking**		
Daily	294	16.3
Occasionally to never	1,505	83.7
**Satisfaction with unpaid work**		
Rather to very dissatisfied	104	5.8
Partly to very satisfied	1,695	94.2
**Depression/depressive mood in the last ** **12** ** months**		
Yes	181	10.1
No	1,618	89.9
**Level of loneliness**		
Increased	135	7.5
Not increased	1,664	92.5
	**Mean**	**SD**
**Basic need autonomy**	3.6	0.7
**Basic need competence**	4.3	0.6
**Basic need relatedness**	4.5	0.5
**Interpersonal trust**	3.6	0.8

SD = standard deviation

**Table 2:** Prevalence of subjectively poor sleep quality by socio-demographic and health variables (n = 781 women, n = 1,018 men). Source: NRW Health Survey 2024

	**Subjectively poor sleep quality**					
	**Female**		**Male**		**Total**	
	**% (n)**	**(95 % CI)**	**% (n)**	**(95 % CI)**	**% (n)**	**(95 % CI)**
**Total**	43.9 (343)	(40.5 – 47.4)	31.4 (320)	(28.6 – 34.3)	36.9 (663)	(34.6 – 39.1)
**Age group (in years)**						
18 – 34	46.9 (46)	(37.3 – 56.8)	29.7 (81)	(24.5 – 35.3)	34.2 (127)	(29.5 – 39.2)
35 – 49	48.8 (102)	(42.1 – 55.6)	31.2 (89)	(26.1 – 36.8)	38.7 (191)	(34.4 – 43.0)
50 – 64	47.2 (127)	(41.3 – 53.2)	34.7 (87)	(29.0 – 40.7)	41.2 (214)	(37.0 – 45.4)
65 +	33.2 (68)	(27.0 – 39.8)	30.1 (63)	(24.2 – 36.6)	31.6 (131)	(27.3 – 36.2)
**Socio-economic status**						
Low	55.0 (82)	(47.0 – 62.9)	40.9 (70)	(33.8 – 48.4)	47.5 (152)	(42.1 – 53.0)
Medium	42.9 (212)	(38.6 – 47.3)	30.9 (189)	(27.3 – 34.6)	36.3 (401)	(33.5 – 39.1)
High	35.5 (49)	(27.9 – 43.7)	26.0 (61)	(20.7 – 31.8)	29.5 (110)	(25.0 – 34.3)
**Smoking**						
Daily	50.0 (64)	(41.4 – 58.6)	36.7 (61)	(29.7 – 44.3)	42.5 (125)	(37.0 – 48.2)
Occasionally to never	42.7 (279)	(39.0 – 46.5)	30.4 (259)	(27.4 – 33.6)	35.7 (538)	(33.4 – 38.2)
**Satisfaction with unpaid work**						
Rather to very dissatisfied	55.4 (31)	(42.3 – 67.8)	54.2 (26)	(40.2 – 67.7)	54.8 (57)	(45.2 – 64.1)
Partly to very satisfied	43.0 (312)	(39.5 – 46.7)	30.3 (294)	(27.5 – 33.3)	35.8 (606)	(33.5 – 38.1)
**Depression/depressive mood in the last ** **12** ** months**						
Yes	67.3 (74)	(58.1 – 75.5)	74.6 (53)	(63.7 – 83.6)	70.2 (127)	(63.2 – 76.5)
No	40.1 (269)	(36.4 – 43.8)	28.2 (267)	(25.4 – 31.1)	33.1 (536)	(30.9 – 35.4)
**Level of loneliness**						
Increased	71.4 (45)	(59.5 – 81.4)	63.9 (46)	(52.4 – 74.3)	67.4 (91)	(59.2 – 74.9)
Not increased	41.5 (298)	(37.9 – 45.1)	29.0 (274)	(26.1 – 31.9)	34.4 (572)	(32.1 – 36.7)

CI = confidence interval

**Table 3:** Means and standard deviations of continuous variables by sleep quality and gender (n = 781 women, n = 1,018 men). Source: NRW Health Survey 2024

	**Subjectively poor sleep quality**			**Subjectively moderate to good sleep quality**		
	**Female (n = 343)**	**Male (n = 320)**	**Total (n = 663)**	**Female (n = 438)**	**Male (n = 698)**	**Total (n = 1,136)**
	**Mean ± SD**	**Mean ± SD**	**Mean ± SD**	**Mean ± SD**	**Mean ± SD**	**Mean ± SD**
**Basic need autonomy**	3.2 ± 0.8	3.4 ± 0.7	3.3 ± 0.7	3.7 ± 0.7	3.7 ± 0.7	3.7 ± 0.7
**Basic needcompetence**	4.0 ± 0.7	4.1 ± 0.7	4.0 ± 0.7	4.4 ± 0.6	4.4 ± 0.5	4.4 ± 0.5
**Basic needrelatedness**	4.4 ± 0.6	4.3 ± 0.6	4.4 ± 0.6	4.6 ± 0.5	4.6 ± 0.5	4.6 ± 0.5
**Interpersonal trust**	3.6 ± 0.8	3.5 ± 0.9	3.5 ± 0.9	3.8 ± 0.8	3.7 ± 0.7	3.7 ± 0.7

SD = standard deviation
